# Divergent electrophysiologic action of dapagliflozin and empagliflozin on ventricular and atrial tachyarrhythmias in isolated rabbit hearts

**DOI:** 10.3389/fcvm.2024.1369250

**Published:** 2024-02-22

**Authors:** Julian Wolfes, Jan Uphoff, Sven Kemena, Felix Wegner, Benjamin Rath, Lars Eckardt, Gerrit Frommeyer, Christian Ellermann

**Affiliations:** Department of Cardiology II, Electrophysiology, University Hospital Münster, Münster, Germany

**Keywords:** SGLT2, dapagliflozin, empagliflozin, langendorff, atrial fibrillation, short QT syndrome, long QT syndrome, arrhythmia

## Abstract

**Background:**

The use of SGLT-2 inhibitors has revolutionized heart failure therapy. Evidence suggests a reduced incidence of ventricular and atrial arrhythmias in patients with dapagliflozin or empagliflozin treatment. It is unclear to what extent the reduced arrhythmia burden is due to direct effects of the SGLT2 inhibitors or is solely a marker of improved cardiac function.

**Methods:**

One hundred five rabbit hearts were allocated to eight groups and retrogradely perfused, employing a Langendorff setup. Action potential duration at 90% of repolarization (APD_90_), QT intervals, effective refractory periods, conduction velocity, and dispersion of repolarization were obtained with monophasic action potential catheters. A model for tachyarrhythmias was established with the I_Kr_ blocker erythromycin for QT prolongation associated proarrhythmia as well as the potassium channel opener pinacidil for a short-QT model. An atrial fibrillation (AF) model was created with isoproterenol and acetylcholine. With increasing concentrations of both SGLT2 inhibitors, reductions in QT intervals and APD_90_ were observed, accompanied by a slight increase in ventricular arrhythmia episodes. During drug-induced proarrhythmia, empagliflozin succeeded in decreasing QT intervals, APD_90_, and VT burden whereas dapagliflozin demonstrated no significant effects. In the presence of pinacidil induced arrhythmogenicity, neither SGLT2 inhibitor had a significant impact on cardiac electrophysiology. In the AF setting, perfusion with dapagliflozin showed significant suppression of AF in the course of restitution of electrophysiological parameters whereas empagliflozin showed no significant effect on atrial fibrillation incidence.

**Conclusion:**

In this model, empagliflozin and dapagliflozin demonstrated opposite antiarrhythmic properties. Empagliflozin reduced ventricular tachyarrhythmias whereas dapagliflozin showed effective suppression of atrial arrhythmias.

## Introduction

The introduction of SGLT2-inhibitors has revolutionized heart failure therapy not only in heart failure with reduced ejection fraction (1, 2) but also in heart failure with preserved ejection fraction (3, 4). The impressive data of recent randomized trials has resulted in a recent update of the ESC guidelines in heart failure (5). Previous studies indicated a reduced incidence of ventricular tachyarrhythmias (VA) and atrial fibrillation (AF) with SGLT2-inhibitor therapy (6). To date, it is unclear whether the antiarrhythmic potency of SGLT2-inhibitors is due to direct interactions with cardiomyocytes or is merely a marker of improved cardiac function. Experimental data on the effect of SGLT2-inhibitors on ventricular and atrial electrophysiology is scarce. Antiarrhythmic effects have been described for dapagliflozin at the ventricular (7–9) level in different animal models. Similarly, ventricular antiarrhythmic effects have been described for empagliflozin in rat (10–12) and mouse models (13, 14) but also rabbit Langendorff models (15), whereas atrial effects are less recorded. Therefore, this study aimed at evaluating acute effects of the two SGLT2-inhibitors dapagliflozin and empagliflozin on atrial and ventricular electrophysiology in an established model.

## Methods

All experimental protocols were approved by the local animal care committee and the local federal institution (Landesamt für Natur, Umwelt und Verbraucherschutz Nordrhein-Westfalen, file number: 81-02.05.50.21.004). The experimental Langendorff whole-heart setup has been extensively described by our group in previous publications (16–19).

In summary, a total of 105 rabbit hearts were obtained after the animals were euthanized by exsanguination. The hearts were aortically clamped into a Langendorff apparatus and retrogradely perfused employing warmed and oxygenated (95% O_2_, 5% CO_2_) KrebsisHenseleit buffer (NaCl 118 mM, NaHCO_3_ 24.88 mM, D-glucose 5.55 mM, KCl 4.70 mM, Na-pyruvate 2 mM, CaCl_2_ 1.80 mM, KH_2_PO_4_ 1.18 mM, MgSO_4_ 0.83 mM). From here on, hearts were divided into two different experimental groups depending on whether the ventricular or atrial electrophysiological effects of SGLT-2 inhibitors were to be studied.

## Experiments on ventricular electrophysiology and tachyarrhythmias

Seven electrophysiological catheters were placed epicardially for the detection of monophasic action potentials and one endocardial catheter was placed in the left ventricle. Furthermore, a pseudo 12 lead ECG was recorded from the warming-bath surrounding the heart. Mechanical AV nodal ablation was performed. Thereafter, the pacing protocol was started:

First, the unstimulated ventricular escape rate of the hearts was determined. Subsequently, pacing was performed with seven different cycle lengths between 900 and 300 ms while the cycle length-dependent monophasic action potentials and QT intervals were determined.

Subsequently, pacing with a short-coupled extrastimulus was performed to determine the effective refractory period (ERP). In addition, repetitive burst stimulations (3 cycles of 1,000 ms at 1 Hz) were used to record ventricular vulnerability. This was followed by perfusion with hypokalemic KHB (K + 1.5 mM) to determine arrhythmia susceptibility in a hypokalemic environment.

Action potential duration at 90% of repolarization (APD_90_) was measured between the fastest upstroke and 90% of repolarization. Spatial dispersion of repolarization was determined by the difference between the maximum and the minimum APD of the eight simultaneously recorded monophasic action potentials. Post-repolarization refractoriness (PRR) was calculated as the difference between ERP and APD_90_.

## Experiments on atrial electrophysiology and tachyarrhythmias

Five epicardial MAP catheters were placed on both atria with two recording MAPs on each atrium and one MAP for stimulation at the interseptal region. The hearts were paced with a cycle-length (CL) of 350, 250, and 150 ms for 1 min to stabilize the action potential before recording 16 consecutive beats at each CL for atrial action potential duration (aAPD_90_) analysis. The atrial effective refractory period (aERP) and atrial post-repolarization refractoriness (aPRR) were determined analogously to the ventricular experiments. Burst pacing (10 cycles of 1,000 ms at 1 Hz) was utilized to evaluate arrhythmia susceptibility.

The 105 hearts were allocated to eight groups: In group 1 (Dapa), 12 hearts (*n* = 12) were treated with 3 µM dapagliflozin. Thereafter, 5 µM dapagliflozin was infused followed by 10 µM dapagliflozin. In group 2 (Ery + Dapa) 12 hearts (*n* = 12) were perfused with 300 µM erythromycin followed by 5 µM dapagliflozin. In group 3 (Pina + Dapa), the 12 hearts (*n* = 12) were perfused with 1 µM pinacidil followed by 5 µM dapagliflozin. Group 4 (IsoACh + Dapa) employed the atrial setup in 13 hearts (*n* = 13). After the determination of the electrophysiological determinants at baseline, hearts were perfused with a combination of isoproterenol (1 µM) and acetylcholine (1 µM) to increase the occurrence of AF. The combination of both drugs has been previously tested on this (20) and other experimental models (21). After 15 min of incubation, the aforementioned pacing protocol was employed again. Afterwards, the hearts were additionally perfused with 3 µM dapagliflozin, and electrophysiological parameters were determined. Each drug intervention underwent a 15-min run-in period before the electrophysiologic study was performed.

In group 5 (Empa), 13 hearts (*n* = 13) were treated with 1 µM empagliflozin. Thereafter, 3 µM empagliflozin was infused followed by 5 µM empagliflozin. In group 6 (Ery + Empa) 17 hearts (*n* = 17) were perfused with 300 µM erythromycin followed by 3 µM empagliflozin. In group 7 (Pina + Empa), the 14 hearts (*n* = 14) were perfused with 1 µM pinacidil followed by 3 µM empagliflozin. Group 8 (IsoACh + Empa) employed the atrial setup in 12 hearts (*n* = 12). Analogous to group 4, after determination of baseline parameters, perfusion with 1 µM IsoACh followed by perfusion with 3 µM Empa was performed.

Electrograms and action potentials were recorded on a multi-channel recorder and digitalized at a rate of 1 kHz with a 12-bit resolution. Variables are shown as mean ± standard deviation. Statistical analyses and graphic visualizations were performed employing Graphpad Prism Version 9. Drug effects on mean APD_90_, QT interval, spatial dispersion of repolarization of all cycle length, effective refractory periods and arrhythmia incidence were analyzed employing repeated measures ANOVA for multiple comparisons. *P* values < 0.05 were considered to be statistically significant.

## Results

### Dapagliflozin

**Figure 1:**
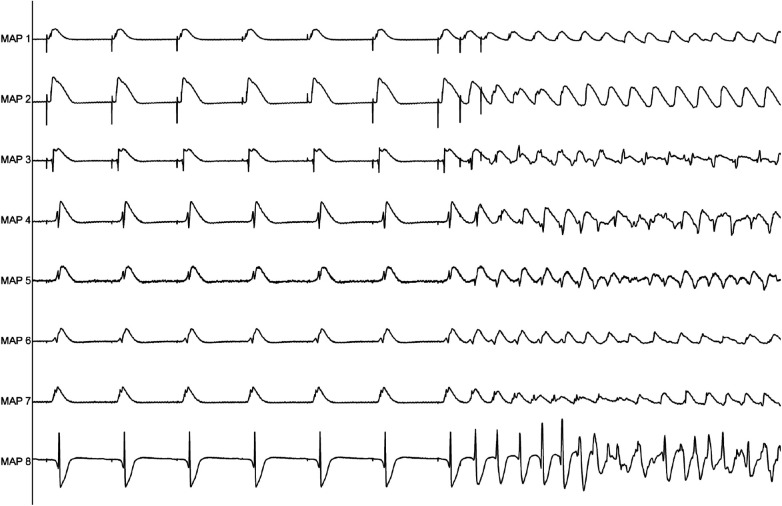
Representative example of ventricular tachycardia arising from programmed ventricular stimulation.

**Figure 2:**
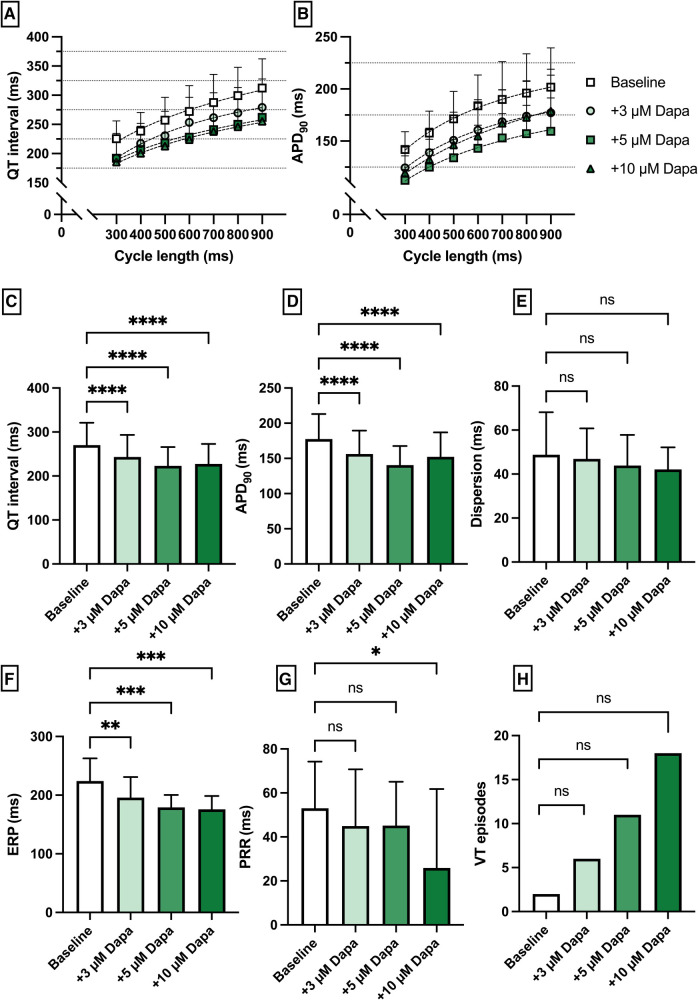
(**A**) Cycle-length dependent QT intervals and (**B**) action potential durations (APD_90_) under baseline conditions (empty square) and after treatment with 3 µM (light green circle), 5 µM (green square) and 10 µM (dark green triangle) dapagliflozin (dapa). (**C**) Overall QT interval and (**D**) APD_90_. Concentration-dependent effect of dapagliflozin on (**E**) spatial dispersion of repolarization, (**F**) effective refractory period (ERP), (**G**) post-repolarization refractoriness (PRR) and (**H**) number of ventricular tachycardia (VT)/fibrillation (VF) induced by programmed ventricular stimulation (* = *p* < 0.05, ** = *p* ≤ 0.01, ** = *p* ≤ 0.001, *** = *p* ≤ 0.0001).

Perfusion with dapagliflozin caused a significant shortening of QT intervals [baseline: 270 ± 51 ms; 3 µM Dapa: 243 ± 50 ms (*p* < 0.05); 5 µM Dapa: 223 ± 43 ms (*p* < 0.05); 10 µM Dapa: 227 ± 46 ms (*p* < 0.05)] and APD_90_ intervals [baseline: 178 ± 36 ms; 3 µM Dapa: 156 ± 33 ms (*p* < 0.05); 5 µM Dapa: 141 ± 27 ms (*p* < 0.05); 10 µM Dapa: 152 ± 35 ms (*p* < 0.05)] in rabbit ventricles, while spatial dispersion of repolarization was not significantly altered. Furthermore, perfusion with dapagliflozin caused a significant shortening of ERP while the PRR shortening reached statistical significance only under 10 µM Dapa. The incidence of ventricular arrhythmia episodes under programmed stimulation (representative example in Figure 1) was tendentially but not significantly increased [baseline: 2 episodes; 3 µM Dapa: 6 episodes (*p* = ns); 5 µM Dapa: 11 episodes (*p* = ns); 10 µM Dapa: 18 episodes (*p* = ns)] (Figure 2).

### Dapagliflozin in a model of QT prolongation associated VA

**Figure 3:**
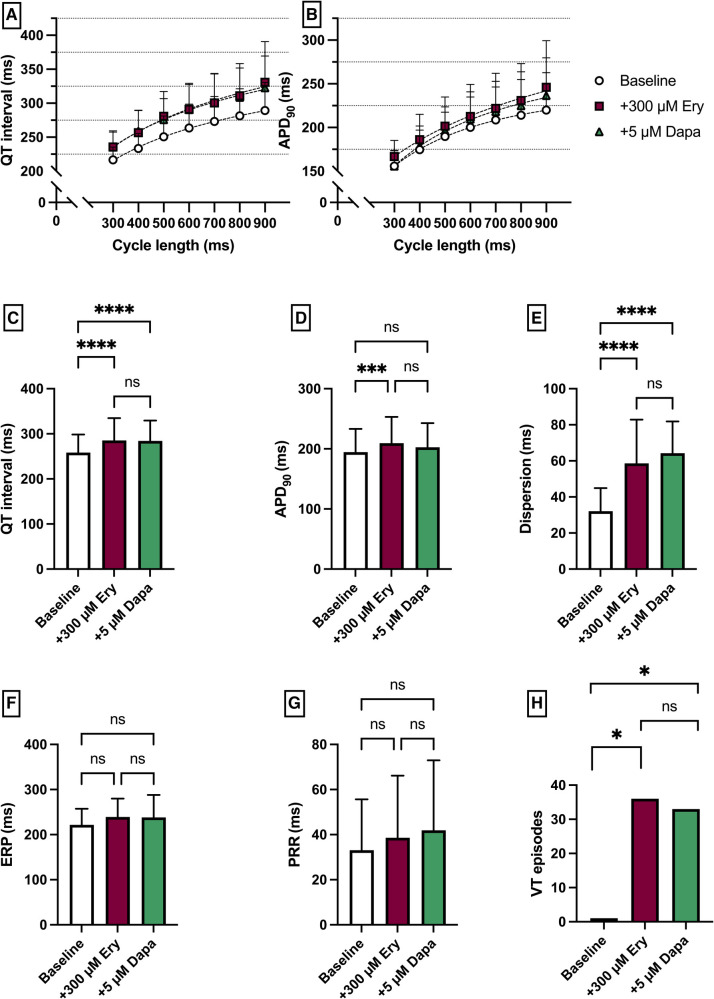
(**A**) Cycle-length dependent QT intervals and (**B**) action potential durations (APD_90_) under baseline conditions (empty circles) and after treatment with 300 µM erythromycin (Ery) (purple square) and additional perfusion with 5 µM (green triangle) dapagliflozin (dapa). (**C**) Overall QT interval and (**D**) APD_90_. Effect of erythromycin and dapagliflozin on (**E**) spatial dispersion of repolarization, (**F**) effective refractory period (ERP), (**G**) post-repolarization refractoriness (PRR) and (**H**) number of ventricular tachycardia (VT)/fibrillation (VF) induced by programmed ventricular stimulation (* = *p* < 0.05, ** = *p* ≤ 0.01, ** = *p* ≤ 0.001, *** = *p* ≤ 0.0001).

Erythromycin (Ery) was employed to induce a medical LQT2 syndrome. The perfusion with Ery caused a significant prolongation of QT interval [baseline: 258 ± 40 ms; + 300 µM Ery: 286 ± 49 ms (*p* < 0.05)] and APD_90_ (baseline: 195 ± 39 ms; + 300 µM Ery: 206 ± 44 ms (*p* < 0.05). The additional perfusion with Dapa did not cause a significant change in QT interval [+5 µM Dapa: 284 ± 45 ms (*p* = 0.97)] and APD_90_ [+5 µM Dapa: 203 ± 40 ms (*p* = 0.15)]. These observations were paralleled by a significant increase in spatial dispersion of repolarization under Ery without significant changes under additional perfusion with Dapa. While ERP and PRR were not significantly altered under both perfusions the arrhythmia susceptibly under programmed stimulation was significantly increased under Ery [baseline: 1 episode; + 300 µM Ery: 36 episodes of torsade de pointes tachycardia (*p* < 0.05); + 5 µM Dapa: 33 episodes (*p* = ns)] (Figure 3).

### Dapagliflozin in a model of QT abbreviation associated VA

**Figure 4:**
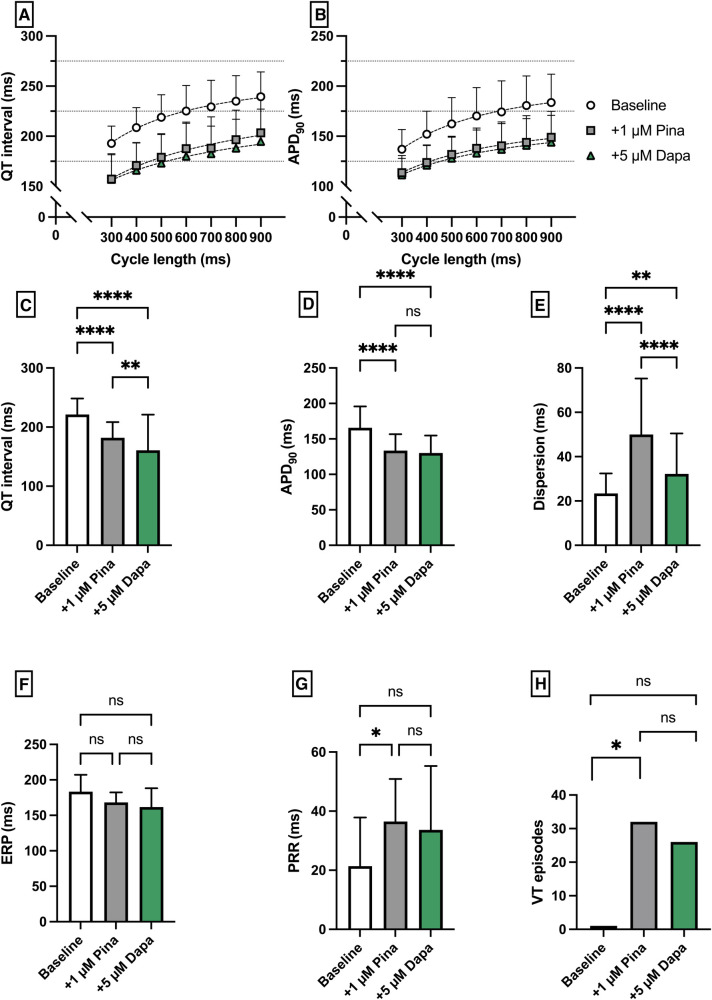
(**A**) Cycle-length dependent QT intervals and (**B**) action potential durations (APD_90_) under baseline conditions (empty circles) and after treatment with 1 µM pinacidil (pina) (grey square) and additional perfusion with 5 µM (green triangle) dapagliflozin (dapa). (**C**) Overall QT interval and (**D**) APD_90_. Effect of pinacidil and dapagliflozin on (**E**) spatial dispersion of repolarization, (**F**) effective refractory period (ERP), (**G**) post-repolarization refractoriness (PRR) and (**H**) number of ventricular tachycardia (VT)/fibrillation (VF) induced by programmed ventricular stimulation (* = *p* < 0.05, ** = *p* ≤ 0.01, ** = *p* ≤ 0.001, *** = *p* ≤ 0.0001).

Pinacidil (Pin) was employed to induce a medical Short QT syndrome. The perfusion with Pina caused a significant shortening of QT interval [baseline: 221 ± 27 ms; + 1 µM Pina: 182 ± 27 ms (*p* < 0.05)] and APD_90_ [baseline: 166 ± 30 ms; + 1 µM Pina: 134 ± 23 ms (*p* < 0.05)]. The additional perfusion with Dapa caused a significant shortening of the QT interval [+5 µM Dapa: 161 ± 60 ms (*p* < 0.05)] while the APD_90_ was not significantly altered [+5 µM Dapa: 130 ± 25 ms (*p* = ns)]. The dispersion was significantly increased under Pina and significantly reduced under Dapa. The incidence of ventricular arrhythmia under programmed stimulation episodes was significantly increased under perfusion with Pina [baseline: 1 episode; + 1 µM Pina: 32 episodes (*p* < 0.05)] and not significantly but tendentially reduced under Dapa [+5 µM Dapa: 26 episodes (*p* = ns)] (Figure 4).

### Dapagliflozin in an atrial fibrillation model

**Figure 5:**
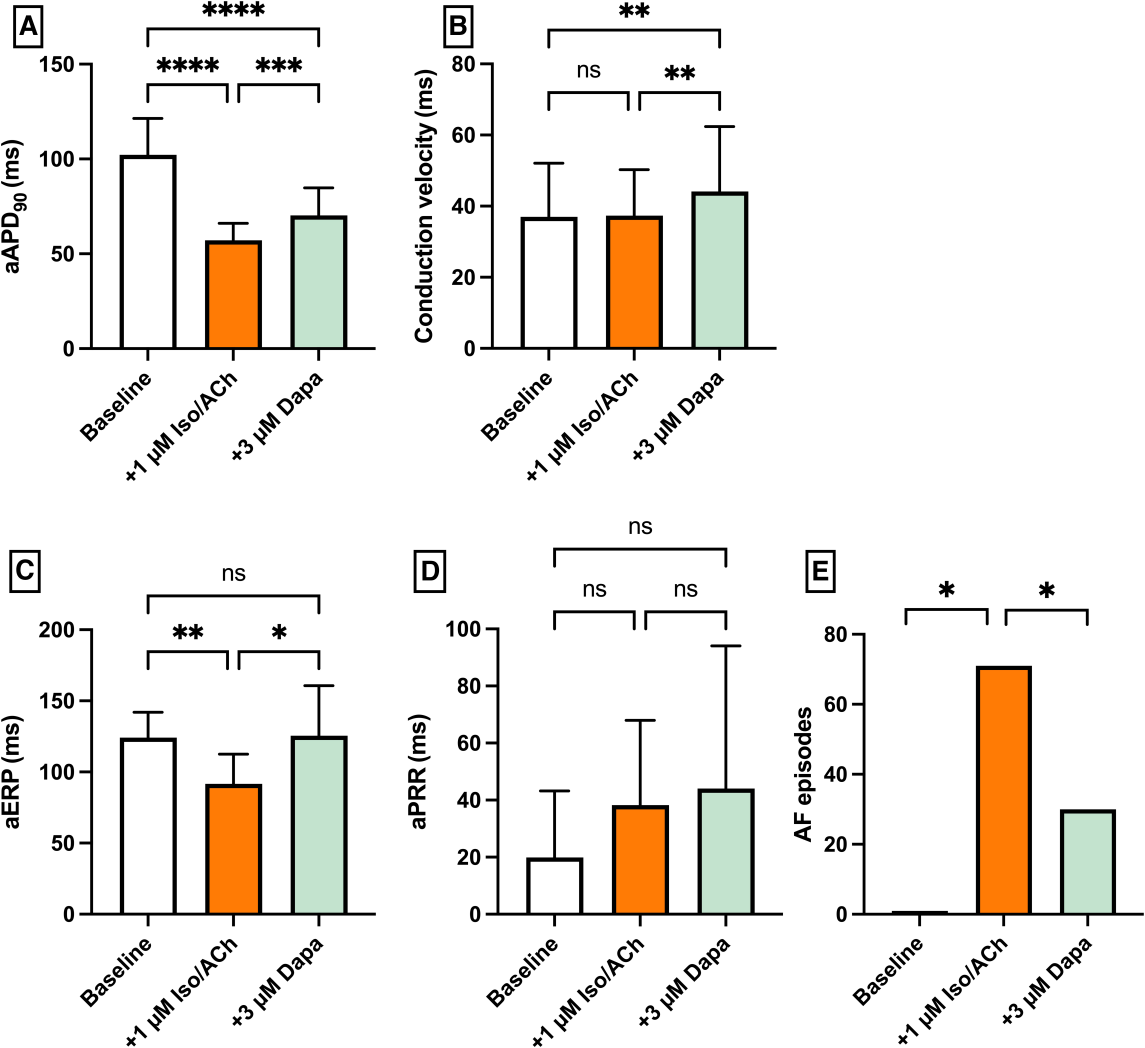
Effect of 1 µM isoproterenol and acetylcholine (Iso/ACh) and 3 µM dapagliflozin (dapa) on (**A**) aAPD_90_, (**B**) conduction velocity, (**C**) atrial effective refractory period (aERP), (**D**) atrial post-repolarization refractoriness (aPRR) and (**E**) atrial fibrillation (AF) episodes (* = *p* < 0.05, ** = *p* ≤ 0.01, ** = *p* ≤ 0.001, *** = *p* ≤ 0.0001).

Perfusion with 1 µM isoproterenol and 1 µM acetylcholine (IsoACh) was employed to raise susceptibility for atrial fibrillation (AF). Perfusion with IsoACh led to a significant shortening of aAPD_90_ [baseline: 102 ± 19 ms; + 1 µM IsoACh: 57 ± 9 ms (*p* < 0.05)] and perfusion with Dapa caused aAPD_90_ prolongation [+3 µM Dapa: 70 ± 15 ms (*p* < 0.05)]. The aERP was significantly shortened under IsoACh and returned to baseline duration under Dapa. The incidence of AF episodes was significantly increased under IsoACh and AF susceptibility was significantly suppressed by Dapa [baseline: 1 episode; 1 µM IsoACh: 71 episodes (*p* < 0.05); + 3 µM Dapa: 30 episodes (*p* < 0.05)] (Figure 5).

### Empagliflozin

**Figure 6:**
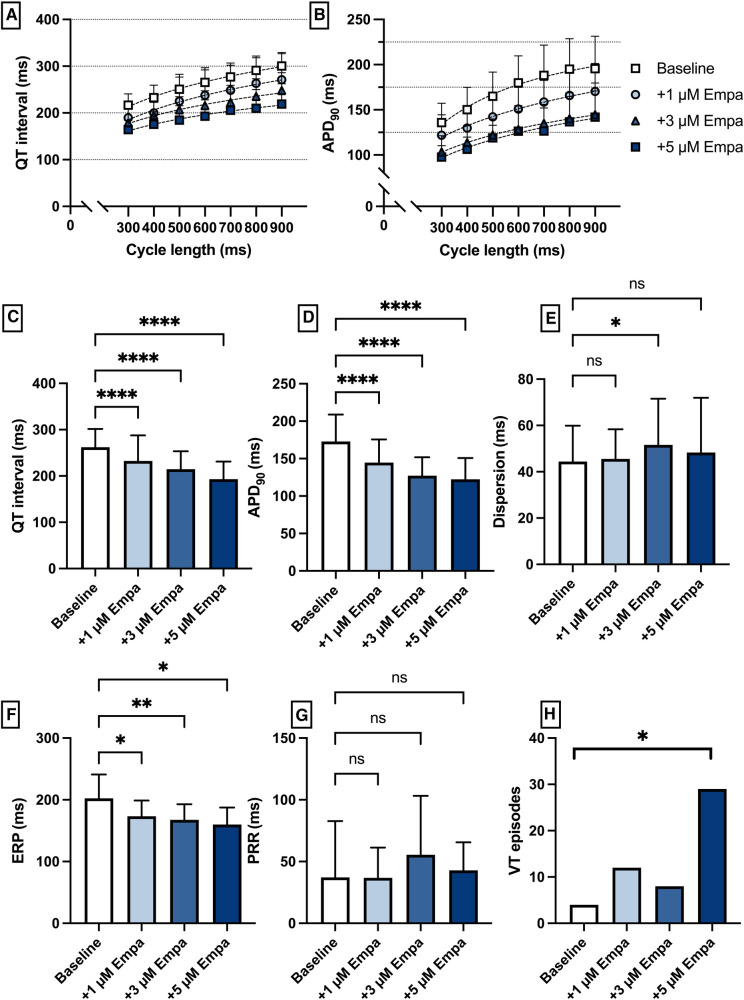
(**A**) Cycle-length dependent QT intervals and (**B**) action potential durations (APD_90_) under baseline conditions (empty square) and after treatment with 1 µM (light blue circle), 3 µM (blue triangle) and 5 µM (dark blue square) empagliflozin (empa). (**C**) Overall QT interval and (**D**) APD_90_. Concentration-dependent effect of empagliflozin on (**E**) spatial dispersion of repolarization, (**F**) effective refractory period (ERP), (**G**) post-repolarization refractoriness (PRR) and (**H**) number of ventricular tachycardia (VT)/fibrillation (VF) induced by programmed ventricular stimulation (* = *p* < 0.05, ** = *p* ≤ 0.01, ** = *p* ≤ 0.001, *** = *p* ≤ 0.0001).

Perfusion with empagliflozin caused a significant shortening of QT intervals [baseline: 262 ± 39 ms; 1 µM Empa: 232 ± 55 ms (*p* < 0.05); 3 µM Empa: 214 ± 39 ms (*p* < 0.05); 5 µM Empa: 193 ± 38 ms (*p* < 0.05)] and APD_90_ intervals [baseline: 172 ± 36 ms; 1 µM Empa: 145 ± 31 ms (*p* < 0.05); 3 µM Empa: 127 ± 25 ms (*p* < 0.05); 5 µM Empa: 122 ± 28 ms (*p* < 0.05)]. Spatial dispersion of repolarization was overall not significantly altered. Furthermore, perfusion with empagliflozin caused a significant shortening of ERP while the PRR was not significantly altered. The incidence of ventricular arrhythmia episodes under programmed stimulation was tendentially increased while only the highest concentration of empagliflozin reached the threshold for statistical significance [baseline: 4 episodes; 1 µM Empa: 12 episodes (*p* = ns); 3 µM Empa: 8 episodes (*p* = ns); 5 µM Empa: 29 episodes (*p* < 0.05)] (Figure 6).

### Empagliflozin in a model of QT prolongation associated VA

**Figure 7:**
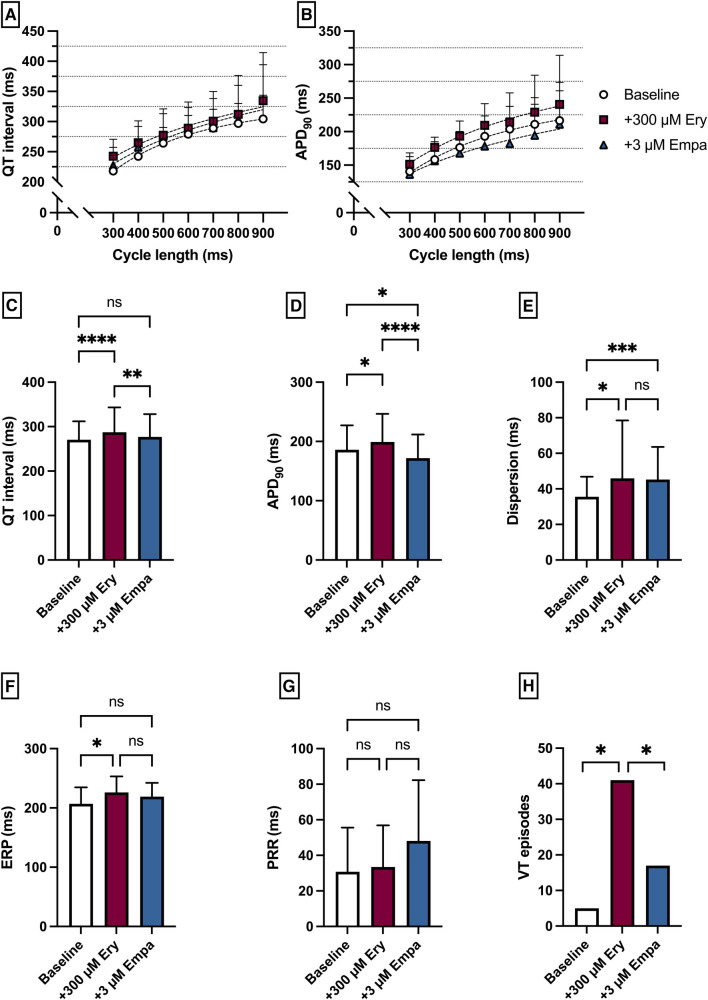
(**A**) Cycle-length dependent QT intervals and (**B**) action potential durations (APD_90_) under baseline conditions (empty circles) and after treatment with 300 µM erythromycin (Ery) (purple square) and additional perfusion with 3 µM (blue triangle) empagliflozin (empa). (**C**) Overall QT interval and (**D**) APD_90_. Effect of erythromycin and empagliflozin on (**E**) spatial dispersion of repolarization, (**F**) effective refractory period (ERP), (**G**) post-repolarization refractoriness (PRR) and (**H**) number of ventricular tachycardia (VT)/fibrillation (VF) induced by programmed ventricular stimulation (* = *p* < 0.05, ** = *p* ≤ 0.01, ** = *p* ≤ 0.001, *** = *p* ≤ 0.0001).

Perfusion with Ery caused a significant prolongation of the QT interval [baseline: 271 ± 42 ms; + 300 µM Ery: 287 ± 56 ms (*p* < 0.05)] and APD_90_ [baseline: 186 ± 41 ms; + 300 µM Ery: 199 ± 48 ms (*p* < 0.05)]. The additional perfusion with Empa caused a significant shortening in QT interval [+3 µM Empa: 277 ± 51 ms (*p* < 0.05)] and APD_90_ [+3 µM Empa: 172 ± 40 ms (*p* < 0.05)]. These observations were paralleled by a significant increase in spatial dispersion of repolarization under Ery without significant changes under additional perfusion with Empa. The ERP was significantly increased under Ery and not significantly altered under Empa. The PRR was not significantly altered under both perfusions. The arrhythmia susceptibly under programmed stimulation was significantly increased under Ery and significantly suppressed by Empa [baseline: 5 episodes; + 300 µM Ery: 41 episodes (*p* < 0.05); + 3 µM Empa: 17 episodes (*p* < 0.05)] (Figure 7).

### Empagliflozin in a model of QT abbreviation associated VA

**Figure 8:**
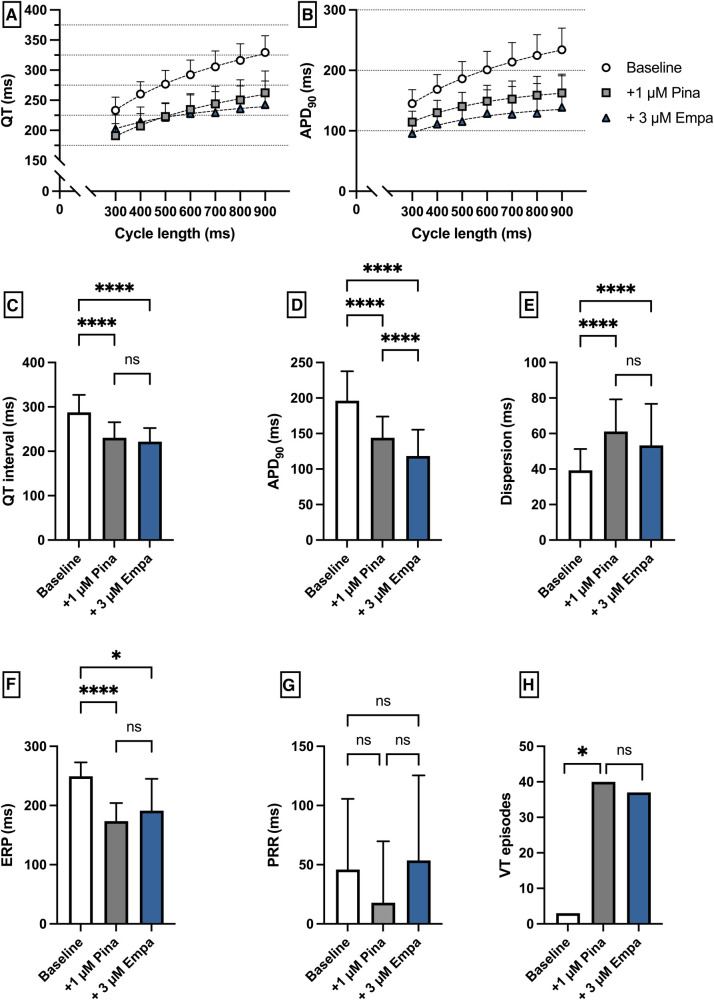
(**A**) Cycle-length dependent QT intervals and (**B**) action potential durations (APD_90_) under baseline conditions (empty circles) and after treatment with 1 µM pinacidil (pina) (grey square) and additional perfusion with 3 µM (blue triangle) empagliflozin (empa). (**C**) Overall QT interval and (**D**) APD_90_. Effect of pinacidil and empagliflozin on (**E**) spatial dispersion of repolarization, (**F**) effective refractory period (ERP), (**G**) post-repolarization refractoriness (PRR) and (**H**) number of ventricular tachycardia (VT)/fibrillation (VF) induced by programmed ventricular stimulation (* = *p* < 0.05, ** = *p* ≤ 0.01, ** = *p* ≤ 0.001, *** = *p* ≤ 0.0001).

Perfusion with Pina caused a significant shortening of QT interval [baseline: 287 ± 39 ms; + 1 µM Pina: 231 ± 35 ms (*p* < 0.05)] and APD_90_ [baseline: 196 ± 42 ms; + 1 µM Pina: 144 ± 30 ms (*p* < 0.05)]. The additional perfusion with Empa caused a significant shortening of the APD_90_ interval [+3 µM Empa: 118 ± 40 ms (*p* < 0.05)] while the QT interval was not significantly altered [+3 µM Empa: 222 ± 31 ms (*p* = ns)]. The dispersion was significantly increased under Pina and not significantly altered under Empa. The incidence of ventricular arrhythmia episodes under programmed stimulation was significantly increased under perfusion with Pina [baseline: 3 episodes; + 1 µM Pina: 40 episodes (*p* < 0.05)] and not significantly altered under Empa [+3 µM Empa: 37 episodes (*p* = ns)] (Figure 8).

### Empagliflozin in an atrial fibrillation model

**Figure 9:**
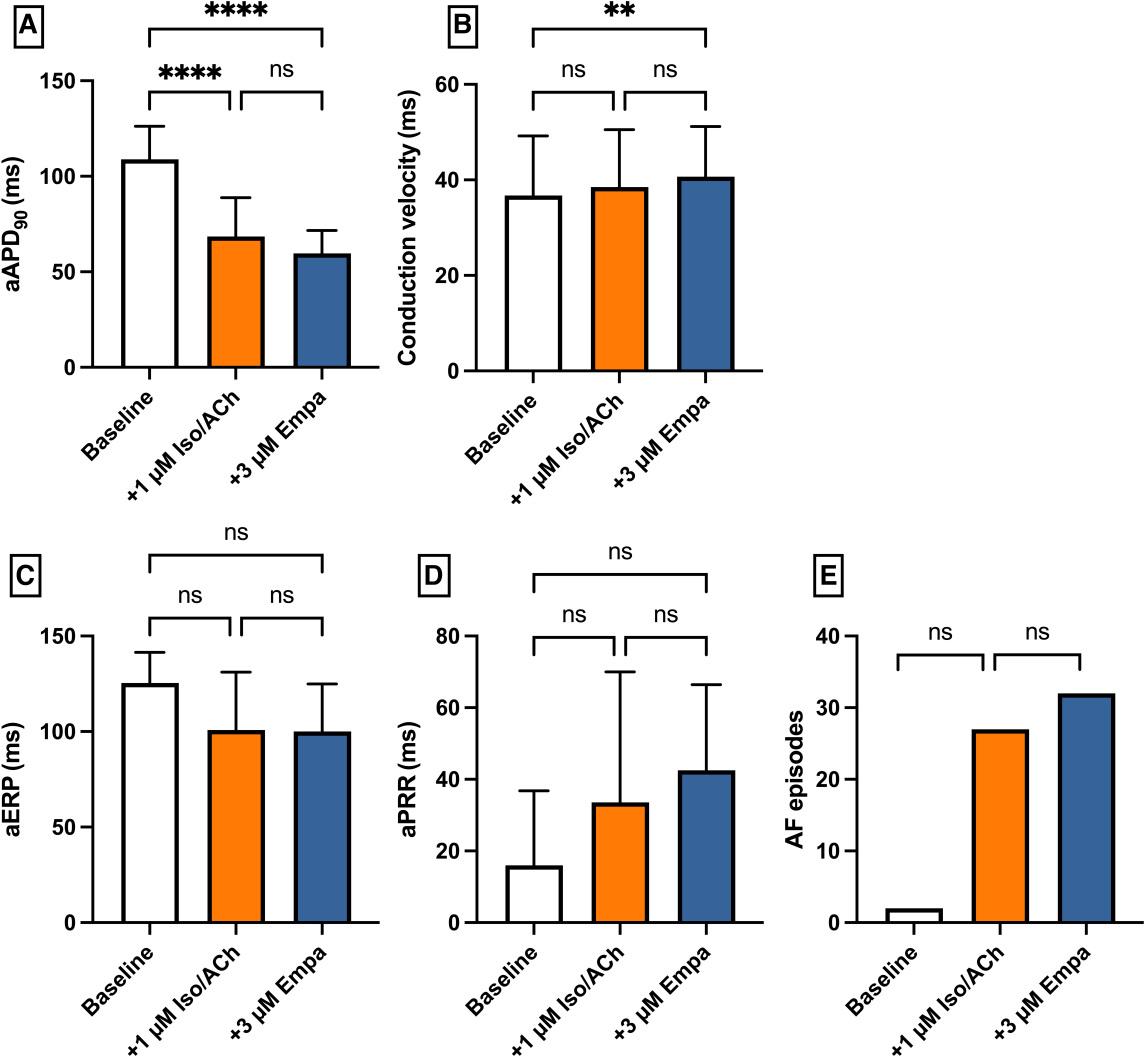
Effect of 1 µM isoproterenol and acetylcholine (Iso/ACh) and 3 µM empagliflozin (empa) on (**A**) aAPD_90_, (**B**) conduction velocity, (**C**) atrial effective refractory period (aERP), (**D**) atrial post-repolarization refractoriness (aPRR) and (**E**) atrial fibrillation (AF) episodes (* = *p* < 0.05, ** = *p* ≤ 0.01, ** = *p* ≤ 0.001, *** = *p* ≤ 0.0001).

Perfusion with IsoACh led to a significant shortening of aAPD_90_ [baseline: 109 ± 17 ms; + 1 µM IsoACh: 69 ± 20 ms (*p* < 0.05)] while perfusion with Empa did not cause a significant change in aAPD_90_ [+3 µM Empa: 60 ± 12 ms (*p* = ns)]. Conduction velocity and eERP were not altered under both perfusions. The incidence of AF episodes was not significantly increased under IsoACh and AF susceptibility was not significantly altered by Empa [baseline: 2 episodes; 1 µM IsoACh: 27 episodes (*p* = ns); + 3 µM Empa: 32 episodes (*p* = ns)] (Figure 9).

## Discussion

The present experimental study on atrial and ventricular electrophysiology and arrhythmias of dapagliflozin and empagliflozin demonstrated the following main results:

(1) Empagliflozin showed antiarrhythmic effects in a model of QT prolongation associated proarrhythmia.
(2) In a model of QT shortening as well as in an AF model, no significant effects of empagliflozin occurred.
(3) In contrast, dapagliflozin showed no antiarrhythmic effects at the ventricular level in the drug-induced LQTS and SQTS. However, significant antiarrhythmic effects were demonstrated in the atrial fibrillation model.

Noteworthy, the concentrations of dapagliflozin and empagliflozin used in this study are consistent with previously published plasma concentrations of dapagliflozin (22) and empagliflozin (23).

## Electrophysiologic effects of dapagliflozin

Perfusion with dapagliflozin significantly shortened cardiac repolarization with shortening of the QT interval and action potential duration. However, the number of VT episodes was not significantly reduced but rather stable with even a trend to more VT episodes. Similar effects of action potential shortening were demonstrated by Durak et al. (8) and Qin et al. (24), who attributed them most likely to an induction of potassium channels. As expected, following the application of the IKr blocker erythromycin, a significant increase in QT interval and APD as well as dispersion occurred, which was accompanied by a significant increase in VA episodes. Perfusion with dapagliflozin did not result in any relevant alteration of APD and QT intervals in this setting, and there was no significant alteration in the number of VA episodes. It is possible that these differences between the previous studies and our observations result from the significantly shorter exposure time of dapagliflozin compared with the previous studies. Perfusion with the potassium channel opener pinacidil resulted in the expected decrease in APD and QT time with a parallel increase in arrhythmia incidence (25, 26). In the same model dapagliflozin showed no overall relevant effects on cardiac electrophysiology.

## Atrial effects of dapagliflozin

In the AF model, dapagliflozin showed an effective reduction in AF episodes. Mechanisms here appear to be the prolongation of the effective refractory period and the restitution of action potential duration. In this study, dapagliflozin's electrophysiologic characteristics resemble those of different class I antiarrhythmic drugs (20, 25, 27). In line with this study, late sodium channel inhibition has been identified as a potential molecular target of different SGLT2 inhibitors in ventricular myocytes (28).

## Ventricular effects of empagliflozin

Perfusion with empagliflozin significantly shortened QT time and action potential duration. However, the number of VA episodes tended to be significantly increased only at high concentrations. Similar effects in terms of action potential shortening were also observed by Xue et al. (13) in a mouse model of myocardial infarction. Furthermore, perfusion with the IKr blocker erythromycin resulted in an increase in QT and APD with a concomitant increase in VA incidence. Here, additive perfusion with empagliflozin resulted in a reduction in QT and APD with a concomitant reduction in VA incidence. The antiarrhythmic effects observed in this model are consistent with observations by Baris et al, who showed that in rats in a sotalol-induced proarrhythmic environment, additive perfusion with empagliflozin led to a decrease in QT (12). Possible mechanisms of this observation in this case could be the previously described suppression of calcium sparks (29) and lowering of intracellular sodium (30). Pathologically prolonged QT is also proarrhythmic in the course of ischemic cardiomyopathies (31) and in animal models of heart failure with preserved ejection fraction (32), providing a possible explanation for direct antiarrhythmic effects of empagliflozin in these conditions. Perfusion with pinacidil resulted in the expected decrease in APD and QT interval with a parallel increase in arrhythmia incidence. The proarrhythmia in a short QT syndrome is usually due to a heterogeneous increase in the repolarizing currents with the resulting increase in dispersion as well as a shortened refractory period with consecutively increased ventricular vulnerability (25, 33). In this setting empagliflozin showed no overall relevant effects on cardiac electrophysiology.

## Atrial effects of empagliflozin

In the atrial fibrillation model, empagliflozin did not show an effective reduction in AF episodes, in contrast to dapagliflozin. Conversely, the group of Shao et al. (11) demonstrated a significant reduction in AF episodes with longer-term empagliflozin use in a rat atrial fibrillation model. This seems most likely to be caused by the different durations of application with transcriptional effects and reduction of atrial fibrosis. Of note, a recent meta-analysis suggests a lower incidence of AF in patients treated with dapagliflozin compared to empagliflozin (34).

## Limitations

Even though the Langendorff model of the isolated rabbit heart is an established model for the investigation of electrophysiological mechanisms (35), its applicability to the human heart remains limited. It should also be noted that variations in baseline values between the groups are most likely due to inter-individual differences and that there were significant differences in AF frequency between the dapagliflozin and empagliflozin groups. Nevertheless, the tendential effects remain largely unaffected by these differences. Furthermore, our model does not allow direct cellular electrophysiology or the observation of transcriptional effects due to the temporal relationship between perfusion and recording. This is of particular importance, as the group of Bers et al. (36) were able to demonstrate transcriptional inhibitory effects of empagliflozin on the late sodium channel influx I_NaL_.

## Conclusion

Empagliflozin showed antiarrhythmic effects in the course of an erythromycin-induced LQT model. In the SQT model as well as the atrial fibrillation model, there was no pronounced effect of perfusion with empagliflozin.

Dapagliflozin showed no antiarrhythmic effects at the ventricular level in the drug-induced LQTS and SQTS. However, significant antiarrhythmic effects were demonstrated in the atrial fibrillation model.

## Data Availability

The datasets presented in this article are not readily available because data available on request from the authors. Requests to access the datasets should be directed to julian.wolfes@ukmuenster.de.
