# Corrigendum: Divergent electrophysiologic action of dapagliflozin and empagliflozin on ventricular and atrial tachyarrhythmias in isolated rabbit hearts

**DOI:** 10.3389/fcvm.2025.1568459

**Published:** 2025-02-24

**Authors:** Julian Wolfes, Jan Uphoff, Sven Kemena, Felix Wegner, Benjamin Rath, Lars Eckardt, Gerrit Frommeyer, Christian Ellermann

**Affiliations:** Department of Cardiology II, Electrophysiology, University Hospital Münster, Münster, Germany

**Keywords:** SGLT2, dapagliflozin, empagliflozin, langendorff, atrial fibrillation, short QT syndrome, long QT syndrome, arrhythmia

A Corrigendum on Divergent electrophysiologic action of dapagliflozin and empagliflozin on ventricular and atrial tachyarrhythmias in isolated rabbit hearts Wolfes J, Uphoff J, Kemena S, Wegner F, Rath B, Eckardt L, Frommeyer G and Ellermann C (2024). Front. Cardiovasc. Med. 11:1369250. doi: 10.3389/fcvm.2024.1369250

In the published article, there was an error in the Funding statement. The author(s) stated that no financial support was received, however the research was funded by Hans und Gertie Fischer-Stiftung, Germany. The correct Funding statement appears below.

